# The Treatment of Internal Derangement of the Knee-Joint

**Published:** 1900-01-13

**Authors:** 


### THE TREATMENT OF INTERNAL DERANGEMENT OF THE KNEE-JOINT.

In dealing with that condition of the knee-joint which is described, almost indifferently, as "internal derangement," "displaced semilunar cartilage," or "subluxation" a very anxious question arises as to treatment. The drift of surgery towards operative interference has shown itself markedly in regard to the treatment of these displacements, and the knowledge that in a certain proportion of such cases operation will be called for and will probably produce a good result, even after the failure of long courses of less heroic treatment, has not unnaturally led to a much more frequent recommendation of operative interference than used to be considered justifiable.

The conclusions arrived at by Mr. Bennett,[1] of St. George's Hospital, from a consideration of a series of 200 such cases, tend, however, in the direction of caution. The cases included in the series were only such as presented the characteristic symptoms of the condition in question, all cases which could be classified under the head of "loose bodies" being excluded. Operation was performed in 27 cases, all males, and in only 15 of these was the semilunar cartilage found to be at fault, the cause of the trouble in the other 12 cases being either small pedunculated bodies or folds of synovial membrane. It is interesting to observe that of the cases in which the semilunar cartilage was involved by far the largest number were in the left knee, whereas in the cases in which other affections were the causes of the mischief the two knees were involved in much more even proportions.

The very important observation is made by Mr. Bennett that in some cases, principally the slighter ones, manipulation not only fails to give relief, but may even cause an increase in the symptoms, the reason being that in such cases there may be nothing to reduce, the symptoms arising neither from the displacement of a semilunar cartilage, nor from the nipping of folds or shreds of synovial membrane between the bones, nor from loose or pedunculated bodies in the joint—the three commonly enumerated causes—but from a bruising of the peripheral edge of a semilunar cartilage and its attachment, without displacement or necessarily loosening, the immediate result of the lesion being a local effusion of blood, reinforced later by inflammatory exudation, which acts as a foreign body between the bones. This is a condition which, although it gives rise to typical symptoms, is incapable of relief by the manipulations which are usually successful in other cases, while it is quite curable by proper treatment and rest. It is to be noted that in such cases it is impossible to completely extend the leg upon the thigh, and that even if by the exercise of force this be done under an anæsthetic the limb will spring back when the pressure is removed. Again, the recovery of such cases under treatment by rest, &c., is gradual—never sudden, as is the case when a foreign body has been removed from between the bones.

The treatment of cases presenting the symptoms of "internal derangement" of the knee-joint may be either—(1) By temporary rest, massage, and exercise; (2) by the use of apparatus; or (3) by operation. In all cases, however, the first thing to do is to endeavour to obtain reduction of the displacement. In the exaggerated cases the attempt must be repeated if it should at first be ineffectual, but in the slight cases above referred to if a careful attempt under an anæsthetic fails it should not be repeated. After this judicious attempt at reduction the proper treatment of all cases, even those in which immediate replacement has been easily effected, is complete rest by fixation of the joint on a light splint, a compress of warm water, or, perhaps better, of lotio plumbi cum opio, being continually applied. This rest may require to be continued from four days to a week, and the less the joint is subjected to movement until all effusion has disappeared the better is the chance of a perfect result. Mr. Bennett says that he is certain, from his own personal experience, and from what he has seen and heard of the results of the practice of others, that "nothing militates against a successful end in these cases so much as the use of exercises, passive or in the gymnasium, immediately after the injury." On the other hand, although no movements of the joint should be commenced until all effusion has gone, massage of muscles and joint without movement cannot be commenced too soon, among its other advantages being that it obviates the tendency to flaccidity of the capsule of the joint which is such a fruitful cause of failure in these cases. When effusion has been removed, and passive movements are commenced, great care must be taken to avoid rotatory movements of the leg, for these tend to interfere with the repair of the injured parts. At the end of three weeks the patient, who till then has been walking about with a "stiff leg," begins ordinary walking exercise. As to the time during which it may be necessary to contine massage, this may be three weeks or six months, or even longer, the important point being that treatment is not to be given up so long as the thigh muscles remain wasted or the capsule of the joint is lax, the lateral movement or "wobbling" of the joint which is associated with such laxity being directly conducive to recurrence of the trouble in the articulation. The cases in which it becomes necessary to permanently employ an apparatus must be taken as failures. Still there are some cases in which their employment may be considered preferable to other measures, but it must always be remembered that ordinary knee-caps are of no use. It is only by preventing rotation of the leg that an instrument can do good. In regard to the question of operation Mr. Bennett says that in the cases in which it is required no operation short of the removal of the offending cartilage is efficient, and he is also of opinion that such removal in these cases does no harm. At the same time he thinks that the number of cases in which operation is ultimately necessary must be very small if the original treatment has been properly managed, and his conclusion is that operative treatment should be reserved for (1) cases in which non-operative measures have conclusively failed; (2) cases in which general flaccidity of the joint is in excess of the other symptoms; (3) cases of expediency, in which an early relief is urgently called for by special circumstances; and (4) grossly neglected cases in which from long-continued inflammation the joint is threatened with pulpy disease. Such cases, however, are rare.

[1] Lancet, Jan. 6.

